# Mitophagy defect mediates the aging‐associated hallmarks in Hutchinson–Gilford progeria syndrome

**DOI:** 10.1111/acel.14143

**Published:** 2024-03-14

**Authors:** Yingying Sun, Le Xu, Yi Li, Shunze Jia, Gang Wang, Xufeng Cen, Yuyan Xu, Zhongkai Cao, Jingjing Wang, Ning Shen, Lidan Hu, Jin Zhang, Jianhua Mao, Hongguang Xia, Zhihong Liu, Xudong Fu

**Affiliations:** ^1^ The First Affiliated Hospital Zhejiang University School of Medicine, and Liangzhu Laboratory of Zhejiang University Hangzhou Zhejiang China; ^2^ Institute of Hematology Zhejiang University Hangzhou Zhejiang China; ^3^ National Clinical Research Center of Kidney Diseases, Jinling Hospital Nanjing University School of Medicine Nanjing Jiangsu China; ^4^ Department of Nephrology, The Children's Hospital, Zhejiang University School of Medicine National Clinical Research Center for Child Health Hangzhou Zhejiang China; ^5^ Center for Stem Cell and Regenerative Medicine, Department of Basic Medical Sciences Zhejiang University School of Medicine Hangzhou China; ^6^ Department of Geriatrics, The First Affiliated Hospital Zhejiang University School of Medicine Hangzhou China

**Keywords:** aging, HGPS, mitophagy, UMI‐77

## Abstract

Hutchinson–Gilford progeria syndrome (HGPS) is a rare and fatal disease manifested by premature aging and aging‐related phenotypes, making it a disease model for aging. The cellular machinery mediating age‐associated phenotypes in HGPS remains largely unknown, resulting in limited therapeutic targets for HGPS. In this study, we showed that mitophagy defects impaired mitochondrial function and contributed to cellular markers associated with aging in mesenchymal stem cells derived from HGPS patients (HGPS‐MSCs). Mechanistically, we discovered that mitophagy affected the aging‐associated phenotypes of HGPS‐MSCs by inhibiting the STING‐NF‐ĸB pathway and the downstream transcription of senescence‐associated secretory phenotypes (SASPs). Furthermore, by utilizing UMI‐77, an effective mitophagy inducer, we showed that mitophagy induction alleviated aging‐associated phenotypes in HGPS and naturally aged mice. Collectively, our results uncovered that mitophagy defects mediated the aging‐associated markers in HGPS, highlighted the function of mitochondrial homeostasis in HGPS progression, and suggested mitophagy as an intervention target for HGPS and aging.

## Graphical abstract

HGPS is a rare disease characterized by premature aging, with limited effective and safe medical interventions due to incomplete understanding of its cellular aging mechanisms. Using patient‐derived cells and mouse models, we demonstrate that mitophagy defects drive aging‐related phenotypes in HGPS by activating the STING‐NF‐ĸB pathway and the senescence‐associated secretory phenotype (SASP). Furthermore, we show that mitophagy induction ameliorates aging‐associated phenotypes of HGPS and natural aging mice, indicating that mitophagy is a potential therapeutic target for both HGPS and aging.

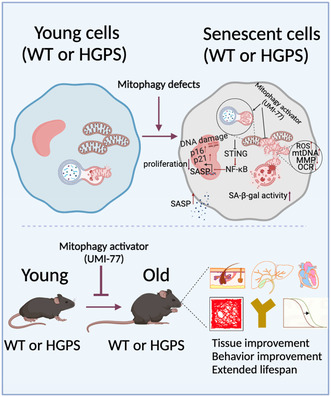

**Abbreviations**
CRISPR: clustered regularly interspaced short palindromic repeats
HGPS: Hutchinson–Gilford progeria syndrome
iPSCs: induced pluripotent stem cells
MSCs: mesenchymal stem cells
NF‐ĸB: nuclear factor kappa‐B
ROS: reactive oxygen species
SASPs: senescence‐associated secretory phenotypes
SA‐β‐Gal: senescence‐associated‐β‐galactosidase
STING: stimulator of interferon genes

## 1 INTRODUCTION

Hutchinson–Gilford progeria syndrome (HGPS), or progeria, is an autosomal dominant childhood disease with an incidence of one in 20,000,000 births (Lamis et al., 2022). Children with HGPS exhibit accelerated aging and multiple aging‐associated phenotypes at both cellular and organ levels, such as accelerated senescence in cells and sarcopenia in muscle (Gordon et al., 2003; Ocampo et al., 2016). The majority of the patients will die from aging‐related diseases, such as myocardial infarction and stroke (Merideth et al., 2008). These features make HGPS a disease model for physiological aging (Ahmed et al., 2018; Ocampo et al., 2016).

Multiple genetic mutations can cause HGPS. The most common mutation (~90%) for HGPS is a point mutation at G608G (GGC > GGT) within exon 11 of the *LMNA* gene (Koblan et al., 2021). This mutation leads to the production of progerin, a truncated variant of Lamin A (Ahmed et al., 2018). The only drug approved by the US Food and Drug Administration (FDA) for HGPS treatment is Lonafarnib, which targets progerin synthesis (Cisneros et al., 2023). However, Lonafarnib causes widespread severe side effects in clinical use, including vomiting, diarrhea, and infection (Gordon et al., 2018). In addition, the clinical impact of Lonafarnib on HGPS patients is limited, and not all patients receiving Lonafarnib treatment exhibit a global reduction in progeria‐associated symptoms, including vascular stiffness, bone structure, and audiological status (Gordon et al., 2012). In this context, novel therapeutic targets and interventions with high efficacy and safety are highly needed to break through the current medical bottleneck of HGPS.

In addition to progerin, aging‐associated hallmarks in HGPS could be targeted to treat the disease (Ocampo et al., 2016). However, the cellular underpinnings of aging hallmarks in HGPS remain largely elusive, resulting in limited therapeutic interventions for the aging phenotypes in HGPS. Previous studies showed that the nuclear lamins are important in mitochondrial function, while the progerin produced by the *LMNA* gene mutation leads to oxidative stress and impaired basal mitochondrial respiration in HGPS cells, and treatment that improves the mitochondrial function delayed the senescence of HGPS cells (Chiang et al., 2022; Maynard et al., 2022; Monterrubio‐Ledezma et al., 2023; Xiong et al., 2016). These results indicate that mitochondria are associated with the aging hallmarks of HGPS. However, how mitochondria affect aging hallmarks in HGPS still remains largely unclear. Mechanistic exploration and in vivo validation are required to reveal the impact of mitochondria in HGPS and HPGS‐associated aging markers.

In this study, by exploiting cellular and mouse models of HGPS, we uncovered that mitophagy, the selective clearance of damaged or dysfunctional mitochondria by lysosomes, mediated the aging‐associated markers in HGPS. Mitophagy is one of the core machineries maintaining mitochondrial function (Palikaras et al., 2015). Our results showed that HGPS cells exhibited defective mitophagy, and the restoration of mitophagy ameliorated aging phenotypes through inhibiting SASP transcription in HGPS models. Furthermore, we revealed that mitophagy induction also alleviated aging‐associated phenotypes in naturally aged mice. These findings highlight the role of mitochondrial recycling in the aging hallmarks of HGPS and suggest that mitophagy is a potential therapeutic target for both HGPS and physiological aging.

## 2 MATERIALS AND METHODS

### 2.1 Cell culture, reagents, and antibodies

Human iPSC cultured with mTeSR™ Plus (Stem cell) were maintained on Matrigel (Corning). Human iPSC‐derived MSCs were cultured in DMEM medium (Cytiva) supplemented with 10% fetal bovine serum (FBS, Vistech), 0.1 mM nonessential amino acids (Gibco), 1% penicillin/streptomycin (Gibco), and 1 ng/mL bFGF (Gibco).

Antibodies used were APC antihuman CD73 (Biolegend, AD2), FITC antihuman CD90 (Biolegend, 5E10), PE antihuman CD105 (Biolegend, SN6h), Anti‐Lamin A/C antibody (Santa, sc‐376,248), TriMethyl‐Histone H3‐K9 Rabbit pAb (Abclonal, A2360), Anti‐Phospho‐Histone H2AX (S139) Antibody (HuaBio, ET1602‐2), Lamin A/C Rabbit mAb (Abclonal, A19524), FITC Goat Anti‐Mouse IgG (H + L) (Abclonal, AS001), TOM20 Rabbit mAb (Abclonal, A19403), NF‐κB p65 antibody (Affinity, AF5006), Phospho‐NF‐κB p65/RelA‐S536‐Rabbit mAb (Abclonal, AP1294), STING/TMEM173 Rabbit mAb (Abclonal, A21051), Anti‐MCL1 Antibody (HuaBio, ET1606‐14), Anti‐Beta‐Actin (HuaBio, EM21002), HRP Goat Anti‐Rabbit IgG (H + L) (Abclonal, AS014), HRP Goat Anti‐Mouse IgG (H + L) (Abclonal, AS003), and Anti‐Progerin antibody (Abcam, ab66587).

The following reagents were used: Polybrene (Yeasen, 40804ES76), H2DCFDA (GLPBIO, GC30006), DAPI (Beyotime, C1002), LysoTracker™ Green (Invitrogen, L7526), Mito‐Tracker Red CMXRos (Beyotime, C1035), and Lipo6000 (Beyotime, C0526).

### 2.2 MSCs generation

About 10 embryoid bodies (EBs) groups were plated on matrigel‐coated 6‐well plates in DMEM medium with 10% FBS, 1% penicillin/streptomycin, 10 ng/mL bFGF, and 5 ng/mL TGFβ (Gibco). Cells were left for about 10 days until differentiation to confluent fibroblast‐like populations occurred.

### 2.3 Flow cytometry

Cells were digested into single cells using trypsin, antibodies were diluited according to the instructions in PBS with 2% FBS, and incubate with the cells on ice for 30 min. The treated cells were analyzed by flow cytometry using DxFLEX (Beckman Coulter), and the data were analyzed with Flow Jo v10.6.2 software.

### 2.4 RNA extraction and quantitative PCR (q‐PCR)

Total RNA was extracted using TRIzol Reagent (CWBio and CW0580), and the cDNA was generated with HiScript II Reverse Transcriptase (Vazyme, R223‐01) according to the instructions. q‐PCR was performed with Taq Pro Universal SYBR qPCR Master Mix (Vazyme, Q712) using QuantStudio 6 Flex (ABI). The data were analyzed by the 2^−ΔΔCt^ method, and relative mRNA expression was normalized to β‐actin. The primer sequences used were as follows: for cells, β‐actin‐fwd (5′‐CACCATTGGCAATGAGCGGTTC‐3′) and β‐actin‐rev (3′‐AGGTCTTTGCGGATGTCCACGT‐5′); p16‐fwd (5′‐CTCGTGCTGATGCTACTGAGGA‐3′) and p16‐rev (3′‐GGTCGGCGCAGTTGGGCTCC‐5′); p21‐fwd (5′‐AGGTGGACCTGGAGACTCTCAG‐3′) and p21‐rev (3′‐TCCTCTTGGAGAAGATCAGCCG‐5′); ki67‐fwd (5′‐GAAAGAGTGGCAACCTGCCTTC‐3′) and ki67‐rev (3′‐TCCTCTTGGAGAAGATCAGCCG‐5′); IL‐1α‐fwd (5′‐TGTATGTGACTGCCCAAGATGAAG‐3′) and IL‐1α‐rev (3′‐CCACAGACCTTCCAGGAGAATG); IL‐1β‐fwd (5′‐CCACAGACCTTCCAGGAGAATG‐3′) and IL‐1β‐rev (3′‐GTGCAGTTCAGTGATCGTACAGG‐5′). for mice, β‐actin‐fwd (5′‐CATTGCTGACAGGATGCAGAAGG‐3′) and β‐actin‐rev (3′‐TGCTGGAAGGTGGACAGTGAGG‐5′); p21‐fwd (5′‐TCGCTGTCTTGCACTCTGGTGT‐3′) and p21‐rev (3′‐CCAATCTGCGCTTGGAGTGATAG‐5′); IL‐1α‐fwd (5′‐ACGGCTGAGTTTCAGTGAGACC‐3′) and IL‐1α‐rev (3′‐ CACTCTGGTAGGTGTAAGGTGC‐5′).

### 2.5 Immunofluorescence staining

Cells plated on coverslips in 24‐well plates were fixed in 4% paraformaldehyde solution (Beyotime) for 10 min and permeabilized with 0.5% Triton X‐100 in PBS for 10 min at room temperature. Cells were incubated with primary antibodies overnight at 4°C after the cells were washed with PBS. The next day, cells were washed with PBS and then incubated with secondary antibodies for 1 h at room temperature. Cell nuclei were stained with DAPI. Images were acquired by a fluorescence microscope (Olympus FV‐3000), and mean fluorescence intensity was calculated by image J.

### 2.6 SA‐β‐gal assay

The SA‐β‐gal staining was performed according to the instructions (Beyotime, C0602). Briefly, cells were fixed for 15 min at room temperature and then incubated with the staining solution for 16 h at 37°C in a CO_2_‐free incubator. Cells were imaged and photographed using a microscope.

### 2.7 ROS detection

Cells were seeded at 35 mm Confocal Dishes (Beyotime). The ROS level of cells was measured by the DCFH‐DA fluorescent probe. Cells were incubated with 10 μM DCFH‐DA in serum‐free media at 37°C for 30 min in the dark. Images were acquired by a fluorescence microscope (Olympus FV‐3000), and mean fluorescence intensity was calculated by image J.

### 2.8 Detection of mitochondrial membrane potential (MMP)

Cells were seeded at 35 mm Confocal Dishes. Cells were stained by JC‐1 according to the instructions (Beyotime, C2006). The relative MMP ratio was calculated as green fluorescence intensity/red fluorescence intensity. Images were acquired by a fluorescence microscope (Olympus FV‐3000), and mean fluorescence intensity was calculated by image J.

### 2.9 Oxygen consumption rates (OCR) measurement

Cells were seeded at a density of 15,000 cells/80 μL well onto XF 96‐well cell culture microplates. According to the instructions, OCR was measured with the Seahorse XFp Cell Mito Stress Test on the Seahorse XFp Analyzer (Agilent, Seahorse Bioscience). OCR was analyzed with Wave software v2.6.1 (Agilent). Compounds used in the assay include: oligomycin (1.5 μM), FCCP (2 μM), and rotenone (0.5 μM).

### 2.10 RNA‐seq analysis

Total RNA of early passage HGPS‐MSCs, late passage HGPS‐MSCs, and UMI‐77‐treated late passage HGPS‐MSCs were extracted for RNA sequencing. The RNA concentration and integrity were assessed using the Bioanalyzer 2100 system (Agilent Technologies, CA, USA). The RNA‐seq libraries were constructed using a VAHTS Universal V6 RNA seq Library Pren Kit (Vazyme) for Illumina and sequenced on an Illumina HiSeq 2500 at Biomarker Technologies. All raw reads were mapped to the human genome (Grch38) with TopHat coupled with Bowtie 2 and default parameters. Transcriptomes were assembled and fragments per kilobase per million reads for each gene were computed with Cufflinks. A cut‐off *p* value <0.05 and absolute values of log_2_‐fold changes greater than 1 were used for differential gene expression analysis. The RNA‐seq data from this publication have been submitted to the GEO database (http://www.ncbi.nlm.nih.gov/geo/) and are accessible through the GEO Series accession number: GSE243251.

### 2.11 Plasmid transfection and viral infection

sgRNA was cloned into lentiCRISPR V2 vector, and the constructed plasmids or nontargeting sgCon plasmid were transfected into HEK‐293 T cells, together with the packaging plasmid psPAX2 and the envelope plasmid pMD2.G (both from Addgene) using PEI. The supernatant was collected for infecting cells. pcDNA3.1‐mt‐Keima was transfected into cells by Lipo6000.

### 2.12 Western blotting

Cells were harvested and solubilized in RIPA buffer containing protease and phosphatase inhibitors. 10 μg protein was electrophoresed on polyacrylamide gels and electroblotted onto PVDF membranes. Proteins were detected by incubation with 1:1000 dilutions of primary antibodies, washed and incubated with Goat anti‐rabbit‐HRP antibodies or Goat anti‐mouse‐HRP antibodies, and detected after incubation with a chemiluminescent substrate.

### 2.13 Animal experiments

All animal studies and experimental procedures were approved by the Animal Care and Use Committee of the animal facility at Zhejiang University. Wild‐type (WT) C57BL/6 mice (12 months old) were purchased from Beijing Vital River Laboratory Animal Technology Co., Ltd. (Beijing, China). F1 heterozygous female and male C57BL/6 mice (*Lmna* ^*G608G/+*^) were kindly provided by Dr. Lidan Hu (Children's Hospital of Zhejiang University). All mice were housed in a pathogen‐free environment at the Animal Experimental Center of Zhejiang University. We selected F2 homozygous mice (*Lmna* ^*G608G/G608G*^) for subsequent experiments. Suspension of UMI‐77 and Sodium Carboxymethyl Cellulose (CMC‐Na) were given to mice by intragastric administration (20 mg/kg, every other day), mice of the control group were only fed CMC‐Na solution. Histological analyses were performed after treatment with UMI‐77 for 5 months of 4‐week‐old *Lmna* ^*G608G/G608G*^ mice or for 7 months of 12‐month‐old WT mice. Behavioral experiments were performed in mice, between 19:00 p.m. and 24:00 p.m., dark phase of mice. The week before the behavioral tests, mice were habituated to a room and a single experimenter by handling in the behavioral room for 5 min a day. After each behavioral stage, the apparatus was thoroughly cleaned with 75% ethanol to prevent olfactory cue bias. To evaluate survival time, *Lmna* ^*G608G/G608G*^ mice were administered with UMI‐77 until the end of mice life.

### 2.14 Open field test

Open field test was performed to test locomotor activity by a white box (40 cm in length, width, and height) made of Plexiglas plate. The mice were placed in the center position at the bottom of the box, allowing them to explore freely for 10 min, and recorded through a camera. Use AlsVision (AniLab Scientific Instruments Co., Ltd., China) to calculate the total movement distance of mice and render trajectory.

### 2.15 Y‐maze test

Y‐maze was performed to detect spatial working memory via spontaneous alternation. The device is a three‐arm maze (40 cm in length, 10 cm in width, and 25 cm in height of every arm) in which each angle is 120°. The mice were placed in the center of the intersection of their three arms and allowed to freely explore the maze for 8 min, and recorded through a camera. Calculate the spontaneous alternation rate by counting the total number of arm movements and alternation times in mice. The number of alternations is the number of times the mice choose to enter an arm that is different from the previous two. Spontaneous alternation rate = number of alternations/(total number of incoming arms−2) × 100%.

### 2.16 Enzyme‐linked immunosorbent assay (ELISA)

Mouse peripheral blood samples were centrifuged at 3000 rpm for 10 min. Serum was collected, aliquoted, and stored at −80°C. The level of IL‐1α and IL‐1β in serum was determined using ELISA kits (Raybiotech) according to the manufacturer's instructions.

### 2.17 Statistical analysis

Statistical and graphical analyses were performed using GraphPad Prism. Results are presented as the mean ± SD. Comparisons were conducted using the two‐tailed student's *t*‐test. Differences were considered to be statistically significant when *p* values were <0.05. All experiments were independently repeated at least three times.

## 3 RESULTS

### 3.1 Establishment of a HGPS cell model

We generated induced pluripotent stem cells (iPSCs) from the peripheral blood mononuclear cells of an HGPS patient (heterozygous *LMNA* c.1824C > T mutation) and differentiated these iPSCs into MSCs (Figure S1A). As expected, these MSCs (referred to as HGPS‐MSCs afterward) express a high level of progerin (Figure S1B).

We next tried to establish the cellular aging phenotypes associated with progeria in HGPS‐MSCs by inducing senescence through continuous passaging. The late‐passage (P7) HGPS‐MSCs exhibited typical cellular aging markers (Lopez‐Otin et al., 2023), including the expression of age‐related stress response genes (*p16* and *p21*) and SASP factors *(IL‐1α* and *IL‐1β*), decreased proliferation capacity, increased senescence‐associated beta‐galactosidase (SA‐β‐gal) activity, increased histone γ‐H2AX (a marker of nuclear DNA double‐strand breaks associated with aging), and aging‐induced epigenetic erosion of H3K9me3 (Figure S1C–J). Of note, HGPS‐MSCs, compared to wild‐type MSCs at the same passage (P7), exhibit elevated cellular markers associated with aging (Figure S1K–M), validating that our replication‐induced senescence of HGPS‐MSCs recapitulates the accelerated aging phenotypes of HGPS patients and can be used in the HGPS study.

### 3.2 Mitophagy defects impair the mitochondrial function of HGPS‐MSCs

It has been reported that mitochondrial function is compromised in HGPS fibroblasts (Chiang et al., 2022; Kreienkamp & Gonzalo, 2020). To validate this phenotype in our HGPS cellular model, we examined the mitochondrial function of HGPS‐MSCs. Our results show that compared to wild‐type MSCs, HGPS‐MSCs exhibit increased reactive oxygen species (ROS) and decreased mitochondrial membrane potential (Figure S2A–C), supporting that HGPS‐MSCs contain compromised mitochondria.

**FIGURE 1:**
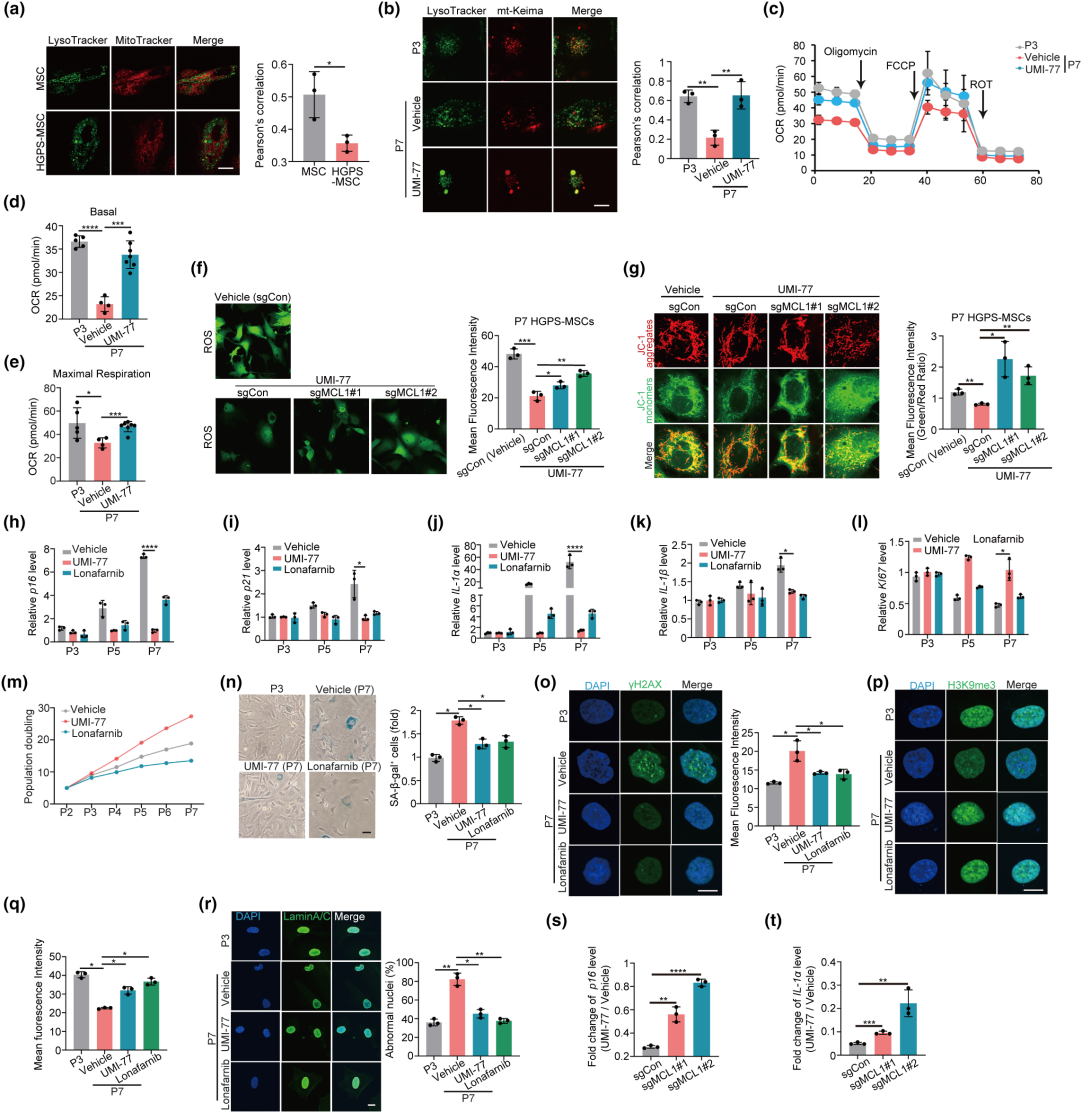
Mitophagy defects contribute to the aging‐associated hallmarks in HGPS‐MSCs. (a) Colocalization of LysoTracker and MitoTracker in MSCs and HGPS‐MSCs at passage 7 (P7) and Pearson's correlation are quantified. Scale bars, 20 μm. (b) Colocalization of LysoTracker and mt‐Keima in HGPS‐MSCs treated with or without UMI‐77 (1 μM) and Pearson's correlation are quantified. Scale bars, 20 μm. (c–e) Cellular oxygen consumption rates (OCR) in early‐passage (P3) (*n* = 5) and late‐passage (P7) HGPS‐MSCs treated with (*n* = 7) or without UMI‐77 (*n* = 4) (c). Basal respiration and maximal respiratory capacity are quantified in (d) and (e) respectively; ROT refers to rotenone. (f) Immunofluorescence analysis and quantification of DCFH‐DA‐based ROS in passage 7 (P7) HGPS‐MSCs treated with or without UMI‐77 (1 μM) after knocking down MCL1. (g) The mitochondrial membrane potential was detected by JC‐1 in P7 HGPS‐MSCs treated with or without UMI‐77 (1 μM) after knocking down MCL1. Scale bars, 20 μm. (h–l) q‐PCR analysis of *p16*, *p21*, *IL‐1α*, *IL‐1β*, and *KI67* mRNA expression in HGPS‐MSCs treated with UMI‐77 (1 μM) or Lonafarnib (12.5 nM). (m) Population doubling calculation in HGPS‐MSCs treated with UMI‐77 (1 μM) or Lonafarnib (12.5 nM). (n) SA‐β‐gal staining and quantification of HGPS‐MSCs treated with UMI‐77 (1 μM) or Lonafarnib (12.5 nM). Scale bars, 20 μm. (o–q) Immunofluorescence analysis and quantification of γH2AX (o) and H3K9me3 (p: immunofluorescence image; q: quantification) in HGPS‐MSCs treated with UMI‐77 (1 μM) or Lonafarnib (12.5 nM). Scale bars, 20 μm. (r**)** Immunofluorescence analysis of Lamin A/C in HGPS‐MSCs treated with UMI‐77 (1 μM) or Lonafarnib (12.5 nM), the percentage of abnormal nuclei was quantified. Scale bars, 20 μm. (s–t) Fold change of *p16* (s) and *IL‐1α* (t) mRNA level between UMI‐77‐treated HGPS‐MSCs and Vehicle‐treated HGPS‐MSCs upon MCL1 perturbation. Data are presented as the mean ± SD. Unpaired *t*‐test was used for statistical analysis. *n* = 3 biological repeats unless otherwise indicated. *p < 0.05, **p < 0.01, ***p < 0.001, ****p < 0.0001.

To uncover the cellular underpinnings of mitochondrial function homeostasis, we examined the involvement of mitophagy in regulating mitochondrial function in HGPS‐MSCs. Interestingly, we found that mitophagy was compromised in HGPS‐MSCs compared to that of normal MSCs, as indicated by the decrease in the colocalization of mitochondria and lysosomes (Figure 1a). Inhibiting mitophagy by Midiv‐1, a selective and transmembrane mitochondrial fission inhibitor that reduces mitophagy (Chang et al., 2022), impairs mitochondrial function of HGPS‐MSCs, as evidenced by the increased ROS production and mitochondria depolarization upon Midiv‐1 treatment (Figure S2D,E), indicating that mitophagy modulates mitochondrial function in HGPS‐MSCs.

Next, we performed rescue assays to further demonstrate that mitophagy defects impair mitochondrial function in HGPS cells. We exploited UMI‐77, a mitophagy activator we previously identified, to restore mitophagy in HGPS‐MSCs (Figure 1b and Figure S2F,G) (Cen et al., 2020). UMI‐77 treatment significantly improves the mitochondrial function of HGPS‐MSCs, as evidenced by the increased basal and maximal respiration levels, decrease of ROS, and the decrease of depolarized mitochondria (Figure 1c–e and Figure S2H–J). In addition, we found that the effect of UMI‐77 on mitochondrial function was mediated by mitophagy. When MCL‐1, the mechanistic target of the mitophagy‐inducing effect of UMI‐77, is perturbed by CRISPR/Cas9 in HGPS‐MSCs (Figure S2K), the inhibitory effect of UMI‐77 on ROS and depolarized mitochondria is decreased (Figure 1f,g). Collectively, these results indicate that mitophagy defects impair mitochondrial function in HGPS cells.

### 3.3 Mitophagy defects mediate the cellular phenotypes associated with aging in HGPS‐MSCs

Mitochondrial dysfunction is intimately linked to aging (Lopez‐Lluch et al., 2018). Many mitochondrial dysfunction drivers, including mitochondrial DNA (mtDNA) mutation, result in accelerated senescence and aging (Kornicka et al., 2017; Wiley & Campisi, 2021). We therefore asked whether mitophagy defects, which drive the mitochondrial dysfunction in HGPS cells, modulate the cellular phenotypes associated with aging in HGPS‐MSCs. We first utilized Midiv‐1 to impair mitophagy in HGPS‐MSCs and found that Midiv‐1 administration enhanced aging‐associated markers in HGPS‐MSCs. The expression of aging markers, including *p16*, *p21*, and SASP factors (*IL‐1α* and *IL‐1β*) in HGPS‐MSCs, are all increased upon Midiv‐1 treatment (Figure S3A–D), while the proliferation of HGPS‐MSCs is decreased by Midiv‐1 administration (Figure S3E). In line with our observation with Midiv‐1, the incubation of bafilomycin A, which impairs mitophagy by interfering with lysosomes, significantly increases cellular aging hallmarks, such as *p16*, *p21*, *IL‐1α*, and *IL‐1β*, and inhibits the expression of proliferation marker (KI‐67) in HGPS‐MSCs (Figure S3F–J). These results indicate that mitophagy defects induce aging‐associated phenotypes in HGPS cells.

To further demonstrate that mitophagy defects contribute to the aging phenotypes in HGPS‐MSCs, we decided to detect whether restoration of mitophagy could rescue aging hallmarks in HGPS‐MSCs. We restored the mitophagy of HGPS‐MSCs via UMI‐77 and found that UMI‐77 administration dramatically decreased aging‐associated makers in HGPS‐MSCs. The expression of cellular aging markers, including *p16, p21*, SASP factors (*IL‐1α* and *IL‐1β*), SA‐β‐gal activity, and γ‐H2AX in HGPS‐MSCs are all decreased upon mitophagy induction, while *KI67* and proliferation capacity of HGPS‐MSCs are increased upon mitophagy activation (Figure 1h–o). Aging‐induced epigenetic erosion of H3K9me3 in HGPS‐MSCs is restored by mitophagy induction (Figure 1p,q). Nuclear membrane shrinkage, induced by progerin, is an important feature and mediator of premature aging in HGPS (Hernandez‐Segura et al., 2018). Mitophagy restoration also reduced the abnormal nuclei proportion of HGPS‐MSCs (Figure 1r), indicating that mitochondrial function may affect nuclear architecture through mitochondria‐nuclear cross‐talks in HGPS cells. Notably, we found that the inhibitory effect of UMI‐77 on the aging markers (*p16* and *IL‐1α*) in HGPS‐MSCs was decreased upon MCL‐1 perturbation (Figure 1s,t), indicating that UMI‐77 delays the aging‐associated phenotypes of HGPS‐MSCs through mitophagy induction. It is worth noting that UMI‐77 was originally known as an apoptosis activator by selectively inhibiting MCL‐1 in tumor cells. To exclude the potential involvement of apoptosis from the role of UMI‐77 on cellular aging, we used a sublethal dose of UMI‐77 throughout the cellular assays (Figure S3K), which induces mitophagy independently of apoptosis (Cen et al., 2020). In addition, we found that Z‐VAD‐FMK (an apoptosis inhibitor) did not decrease the effect of UMI‐77 on inhibiting the aging‐associated phenotypes of HGPS‐MSCs (Figure S3L–P), indicating that UMI‐77 modulates cellular aging hallmarks independently of apoptosis. Altogether (Figure S3Q–V), our results suggest that the mitophagy defects mediate the cellular phenotypes associated with aging in HGPS‐MSCs.

Interestingly, although Lonafarnib (FDA‐approved HGPS drug) alleviates multiple aging phenotypes of HGPS‐MSCs (Figure 1h–k), it does not increase *KI67*, a marker of cellular proliferation, in late‐passage HGPS‐MSCs and even decreases the proliferation capacity of HGPS‐MSCs (Figure 1l,m). This is because the inhibitory effect of Lonafarnib on farnesylation affects targets other than progerin and impairs cellular viability and proliferation (Arnold et al., 2021; Yang et al., 2010). On the contrary, UMI‐77 increases *KI67* and improves the proliferation ability of HGPS‐MSCs (Figure 1l,m), highlighting less toxicity of mitophagy induction on HGPS‐MSCs compared to that of Lonafarnib.

### 3.4 Mitophagy mediates the aging markers of HGPS‐MSCs through the STING‐NF‐κB pathway and the SASP transcription

**FIGURE 2:**
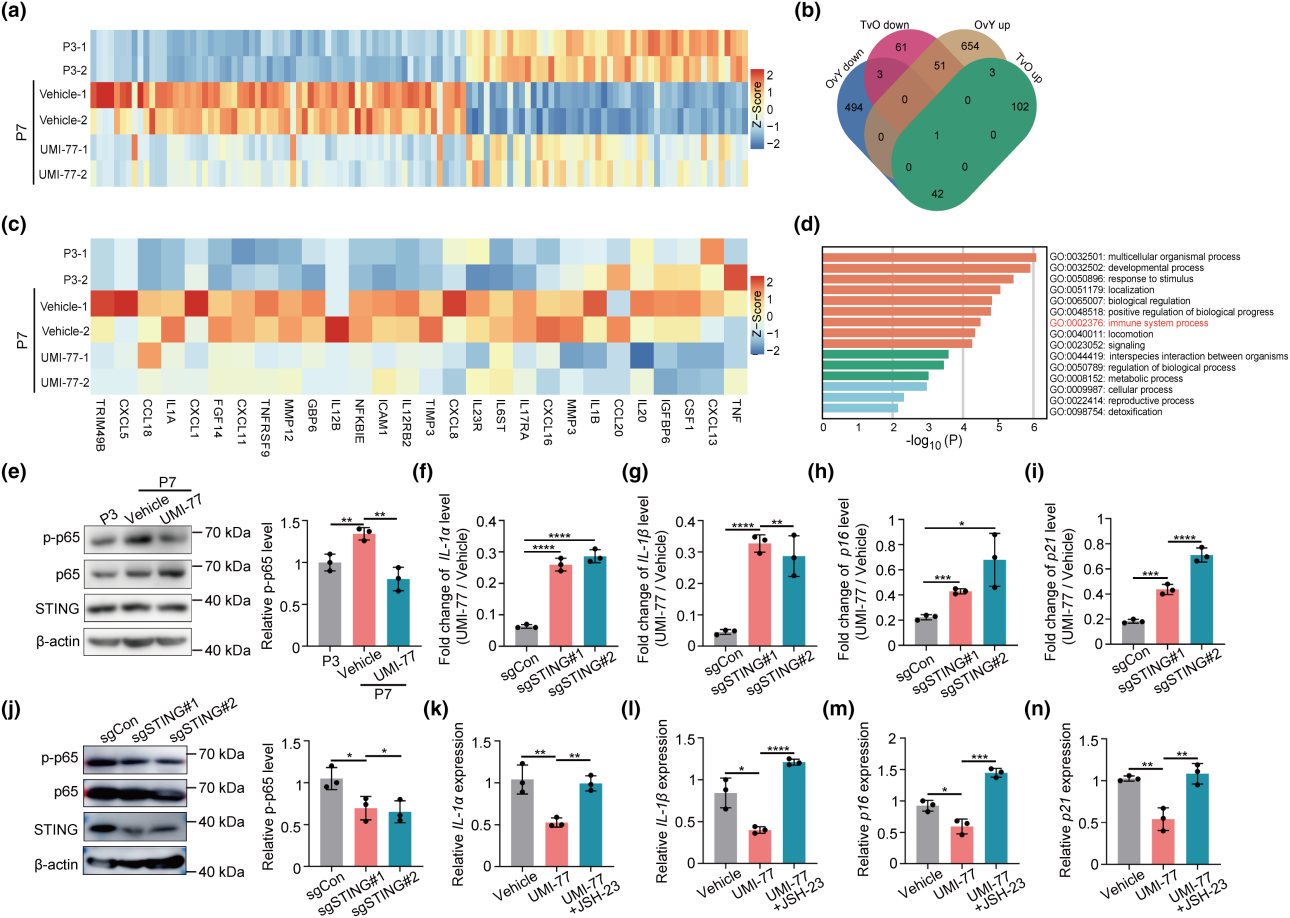
Mitophagy mediates the aging‐associated markers of HGPS‐MSCs through the cGAS‐STING‐NF‐kB pathway and downstream SASP transcription. (a) Column clustered heatmap showing all genes that are differentially expressed (FDR ≤5%, absolute log2 ≥ 1) between early passage HGPS‐MSCs (P3), late passage HGPS‐MSCs (P7), and UMI‐77‐treated late passage HGPS‐MSCs. (b) Venn diagram showing the overlap of the differentially expressed genes (FDR ≤5%, absolute log2 ≥ 1) between early passage HGPS‐MSCs (P3) and late passage HGPS‐MSCs (P7) and between late passage HGPS‐MSCs (P7) and UMI‐77‐treated late passage HGPS‐MSCs. Y: P3 HGPS‐MSCs, O: P7 HGPS‐MSCs, T: UMI‐77‐treated P7 HGPS‐MSCs. (c) Top‐level Gene Ontology biological processes of the “fully reversed” genes, as defined by metascape. (d) Column clustered heatmap showing all SASP genes that are differentially expressed between early passage HGPS‐MSCs (P3), late passage HGPS‐MSCs (P7), and UMI‐77‐treated late passage HGPS‐MSCs. (e) Western blot analysis and quantification of p65 phosphorylation in early passage HGPS‐MSCs (P3), late passage HGPS‐MSCs (P7), and UMI‐77‐treated late passage HGPS‐MSCs. (f–i) Fold change of *IL‐1α*, *IL‐1β*, *p16*, and *p21* mRNA level between UMI‐77‐treated HGPS‐MSCs and Vehicle‐treated HGPS‐MSCs upon STING perturbation. (j) Western blot analysis and quantification of p65 phosphorylation in HGPS‐MSCs upon STING perturbation. (k–n) q‐PCR analysis of *p16*, *p21*, *IL‐1α*, and *IL‐1β* mRNA expression in HGPS‐MSCs upon indicated administration. UMI‐77, 1 μM; JSH‐23, 10 μM. Data are presented as the mean ± SD. Unpaired *t*‐test was used for statistical analysis. *n* = 3 biological repeats. *p < 0.05, **p < 0.01, ***p < 0.001, ****P < 0.0001.

Mitochondrial homeostasis is associated with progeria phenotypes in HGPS cells, but the mechanistic link remains largely elusive (Cisneros et al., 2023). Our results demonstrate that mitophagy defects contribute to the aging hallmarks in HGPS cells. To further elucidate the underlying mechanisms, we perform transcriptomic analysis on early‐passage HGPS‐MSCs (P3), late‐passage HGPS‐MSCs (P7), and UMI‐77‐treated late‐passage (P7) HGPS‐MSCs. The analysis of differentially expressed genes (DEGs) among these three groups revealed that UMI‐77 treatment reversed the expression of the DEGs induced by the senescence of HGPS‐MSCs (Figure 2a). These reversed DEGs are functionally enriched in the immune system process and consist of many SASP genes (Figure 2b–d), indicating that mitophagy inhibits the expression of SASP genes in HGPS cells.

Mitochondria can directly influence inflammation (Poor & Chandel, 2023). The release of mtDNA from compromised mitochondria can activate STING and increase the transcription of the downstream inflammation genes by activating IRF3 and NF‐ĸB (Yu et al., 2020). Interestingly, the SASP genes affected by UMI‐77, which are majorly chemokines and interleukins, are transcriptionally controlled by NF‐ĸB (Figure 2c). These results suggest that mitophagy may affect SASP genes in HGPS cells through the STING‐NF‐ĸB pathway.

To verify the hypothesis, we first detected the impact of mitophagy on STING‐NF‐ĸB signaling in HGPS‐MSCs. The phosphorylation of p65, a main NF‐ĸB subunit (Lawrence, 2009), is increased in the late‐passage HGPS cells, and UMI‐77 administration reverses the activation of NF‐ĸB (Figure 2e), indicating that mitophagy induction inhibits the STING‐NF‐ĸB signaling in HGPS cells. Next, we generated STING‐deficient HGPS‐MSCs through CRISPR/Cas9 (Figure S4A). STING deficiency reduces the inhibitory effect of UMI‐77 on the expression of SASP factors (*IL‐1α* and *IL‐1β*), *p16*, and *p21* (Figure 2f–i), indicating that UMI‐77 modulates cellular aging phenotypes through STING. In addition, the phosphorylation of p65 is inhibited after STING perturbation, which suggests that NF‐ĸB is regulated by STING in HGPS‐MSCs (Figure 2j). Furthermore, JSH‐23, an inhibitor of p65 phosphorylation, normalizes the inhibitory effect of UMI‐77 on cellular aging‐related markers (Figure 2k–n). These results collectively indicate that mitophagy modulates aging hallmarks in HGPS‐MSCs via the STING‐NF‐ĸB pathway and the downstream SASP transcription. Interestingly, although STING is often activated by cGAS, we found that cGAS deficiency did not normalize the effect of UMI‐77 on the expression of *p16* and SASP factors (*IL‐1α* and *IL‐1β*) (Figure S4B–E), indicating that UMI‐77 does not modulate STING by cGAS in HGSP‐MSCs.

### 3.5 Mitophagy induction ameliorates the premature aging phenotypes of HGPS mice

After demonstrating the effect of mitophagy on aging hallmarks in HGPS cells, we set out to investigate whether mitophagy mediates premature aging phenotypes in vivo. We utilized *Lmna* ^G608G/G608G^ mice, a well‐established HGPS mice model which expresses progerin and exhibits multiple progeria‐associated syndromes (McKenna et al., 2014; Sagelius et al., 2008), for the in vivo investigation. The schematic depiction of the UMI‐77 administration plan is shown in Figure S5A. Notably, mitochondrial function is likely to be damaged in the *Lmna* ^G608G/G608G^ mice (referred to as HGPS mice, Figure S5B), supporting that mitochondrial function and homeostasis are associated with progeria phenotypes of HGPS mice.

**FIGURE 3:**
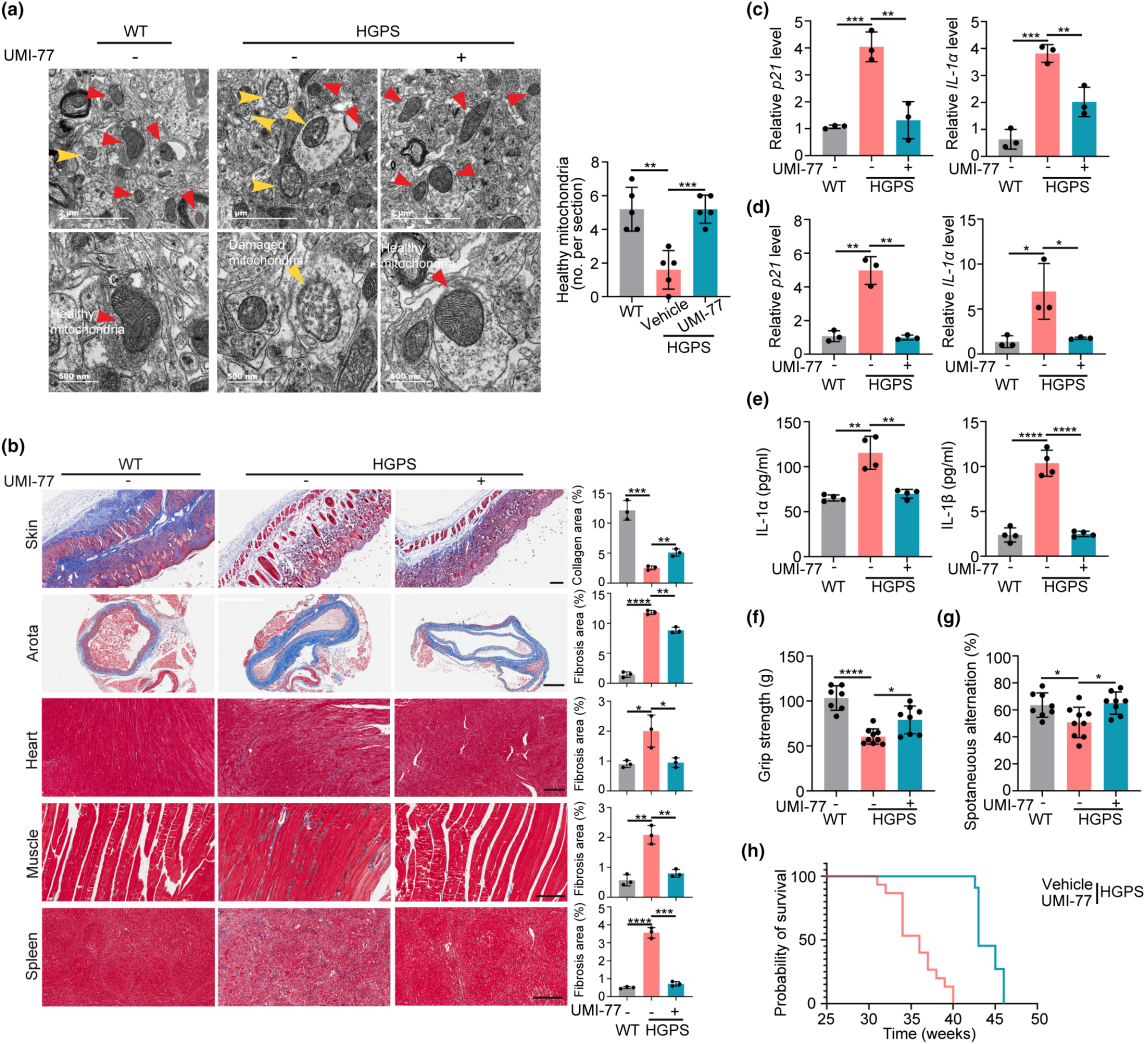
Mitophagy activator UMI‐77 ameliorates the premature aging phenotypes of HGPS (*Lmna* ^*G608G/G608G*^) mice. (a) Representative TEM images (left) and quantification (right) of healthy mitochondria numbers in the brain of WT and HGPS mice treated with or without UMI‐77. *n* = 3 biological repeats. (b) Masson staining of skin, aorta, heart, muscle, and spleen in WT and HGPS mice treated with or without UMI‐77. Scale bars, 200 μm. *n* = 3 biological repeats. (c, d) q‐PCR analysis of *p21* and *IL‐1α* mRNA expression of the heart (c) and spleen (d) in WT and HGPS mice treated with or without UMI‐77. n = 3 biological repeats. (e) ELISA analysis of IL‐1α and IL‐1β secretion in serum of WT and HGPS mice treated with or without UMI‐77. *n* = 3 biological repeats. (f) Forelimb grip strength analysis of WT (*n* = 7) and HGPS mice treated with (*n* = 8) or without UMI‐77 (*n* = 9). (g) Y maze spontaneous alternation showed spatial working memory of WT (*n* = 8) and HGPS mice treated with (*n* = 8) or without UMI‐77 (*n* = 9). (h) Kaplan–Meier survival plot of HGPS mice treated with (*n* = 11) or without UMI‐77 (*n* = 15); The median lifespan of HGPS mice is 252 days (Vehicle) and 301 days (UMI‐77); The maximal lifespan of HGPS mice is 284 days (Vehicle) and 324 days (UMI‐77). Data are presented as the mean ± SD. Unpaired *t*‐test was used for statistical analysis. *p < 0.05, **p < 0.01, ***p < 0.001, ****p < 0.0001.

To explore whether mitophagy modulates the mitochondrial defects and the aging‐associated phenotypes in HGPS mice, we exploited UMI‐77 treatment to test the hypothesis. UMI‐77 was given to 4‐week‐old mice by intragastric administration every other day. UMI‐77 administration restores the morphology of mitochondria of HGPS mice and also increases the basal and maximal respiration levels in liver cells of mice (Figure 3a, Figure S5C–E), indicating that mitophagy modulates the mitochondria function in HGPS mice. Accompanied by the restoration of mitochondria, aging‐associated histological changes in HGPS mice are dramatically restored upon UMI‐77 treatment. For instance, we observed that UMI‐77 slowed down the loss of collagen fibers in the skin of HGPS mice through the classic staining method of connective tissue fibers, Masson staining (Figure 3b). Besides, UMI‐77 reduces fibrosis in tissues such as the aorta, heart, muscles, and spleen of HGPS mice (Figure 3b). At the molecular level, UMI‐77 treatment also restores progeria‐induced aging phenotypes. We found that the expression of *p21* and *IL‐1α* significantly increased in the heart and spleen of HGPS mice compared to that of wild‐type (WT) mice, and UMI‐77 treatment reduced the increase of these aging markers in HGPS mice (Figure 3c,d). In alignment with the inhibitory effect of mitophagy on SASP in HGPS cells, the induction of mitophagy via UMI‐77 likewise led to a reduction in the secretion of SASP factors, namely IL‐1α and IL‐1β, within the serum of HGPS mice (Figure 3e).

In addition, UMI‐77 improves the overall health of HGPS mice. For example, UMI‐77 increases the hair and weight in HGPS mice (Figure S5F–H). Regarding the behavior test, we found that UMI‐77 increased the skeletal muscle strength, spatial working memory, and mobility of HGPS mice (Figure 3f,g, and Figure S5I). Furthermore, UMI‐77 treatment results in a significant 20% increase in the median lifespan and a 15% increase in the maximal lifespan of HGPS mice (Figure 3h). Collectively, our results indicate that mitophagy modulates the mitochondrial defects and premature aging phenotypes in HGPS mice, and mitophagy induction can improve the health and extend the lifespan of HGPS mice.

### 3.6 Mitophagy induction alleviates the aging‐associated phenotypes in wild‐type cells and mice

**FIGURE 4:**
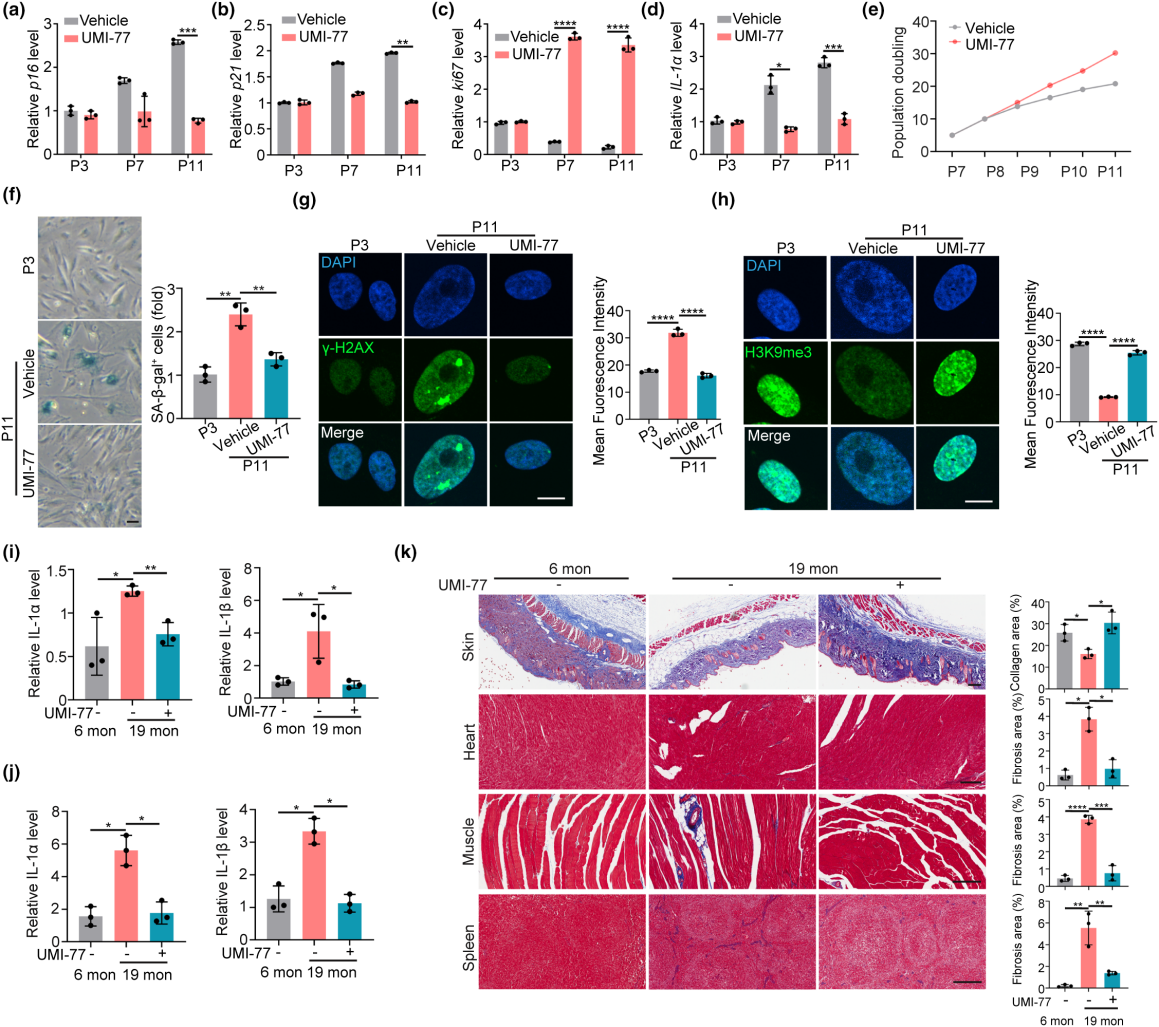
Mitophagy induction alleviates the aging phenotypes in wild‐type cells and mice. (a–d) q‐PCR analysis of *p16*, *p21*, *KI67*, and *IL‐1α* mRNA expression in MSCs treated with or without UMI‐77 (1 μM). (e) Population doubling calculation in MSCs treated with or without UMI‐77 (1 μM). (f) SA‐β‐gal staining and quantification of MSCs treated with or without UMI‐77 (1 μM). Scale bars, 20 μm. (g, h) Immunofluorescence analysis and quantification of γH2AX (g) and H3K9me3 (h) in MSCs treated with or without UMI‐77 (1 μM). Scale bars, 20 μm. (i, j) q‐PCR analysis of *IL‐1α* and *IL‐1β* mRNA expression of the spleen (i) and liver (j) in 6‐month and 19‐month male mice treated with or without UMI‐77. (k) Masson staining of skin, heart, muscle, and spleen in 6‐month and 19‐month male mice treated with or without UMI‐77. Scale bars, 200 μm. Data are presented as the mean ± SD. Unpaired *t*‐test was used for statistical analysis. *n* = 3 biological repeats. *p < 0.05, **p < 0.01, ***p < 0.001, ****p < 0.0001. 6 mon refers to 6 months, and 19 mon refers to 19 months.

HGPS is considered as a model for accelerated physiological aging since it shares multiple aging hallmarks (Harhouri et al., 2017). The efficacy of mitophagy induction via UMI‐77 in alleviating aging hallmarks in HGPS thus inspires us to explore the effect of mitophagy in physiological aging. Firstly, we examined the effect of UMI‐77 on the cellular phenotypes associated with aging. Replication‐induced senescence model in normal MSCs was used. We found that UMI‐77 treatment decreased multiple aging‐related markers (including *p16*, *p21*, *IL‐1α*, SA‐β‐gal activity, and γ‐H2AX) and rescued the proliferation capacity and aging‐induced epigenetic erosion of H3K9me3 in MSCs (Figure 4a–h). These results collectively indicate that UMI‐77 ameliorates cellular hallmarks of aging in wild‐type MSCs.

Next, we examined the effect of UMI‐77 on delaying aging in the mouse model. UMI‐77 was given to 12‐month‐old wild‐type mice (middle‐aged mice) (Flurkey et al., 2007) by intragastric administration every other day for 12 months (Figure S6A). The effect of UMI‐77 on aging phenotypes was detected on 19‐month‐old or 24‐month‐old aging mice. We found that UMI‐77 increased the basal and maximal respiration levels in the liver cells of mice (Figure S6B–D). We also observed that UMI‐77 dramatically inhibited the expression of *p16* and *IL‐1α* in the spleen and liver in mice (Figure 4i,j). In addition, UMI‐77 prevents the loss of collagen fibers in the skin and decreases fibrosis in the heart, muscle, and spleen of aging mice (Figure 4k). Finally, UMI‐77 increased the skeletal muscle strength and mobility (Figure S6E,F). These results provide evidence proving that UMI‐77 exhibits an antiaging effect for physiological aging and support that mitophagy could be exploited in antiaging interventions.

## 4 DISCUSSION

HGPS is a rare genetic disorder majorly induced by *LMNA* mutation (Ullrich & Gordon, 2015). This disease is characterized by accelerated aging and currently lacks efficient and safe medical interventions (Strandgren et al., 2017). This is partially because the cellular and molecular underpinnings of aging‐associated phenotypes in HGPS have yet to be fully elucidated, and the validated therapeutic target for HGPS is limited (Harhouri et al., 2018). For instance, although fibroblasts from HGPS patients exhibited compromised mitochondria, the role of mitochondrial function in HGPS or HGPS treatment was not fully elucidated (Rivera‐Torres et al., 2013). Here, we report that mitophagy defects impair mitochondrial function and contribute to the aging‐associated markers in HGPS cell and mouse models, and pharmacological induction of mitophagy serves as an effective and safe intervention in alleviating aging phenotypes associated with HGPS.

The most common aging‐related symptoms in HGPS patients include scleroderma, myatrophy, atherosclerosis, and myocardial infarction (Lamis et al., 2022; Ullrich & Gordon, 2015). According to our results in vivo, UMI‐77 slows down the loss of hair and collagen fibers in the skin and increases the thickness of the dermis in *Lmna* ^*G608G/G608G*^ mice. Concurrently, UMI‐77 significantly inhibits fibrosis of the aorta, heart, and muscles in *Lmna* ^*G608G/G608G*^ mice, which is closely related to cardiovascular disease and myatrophy pathology, respectively. These data indicate that mitophagy induction effectively alleviates common clinical syndromes associated with HGPS patients in animal models, which supports the next step of clinical application of mitophagy activators, such as UMI‐77, for HGPS patients.

Lonafarnib is currently the only FDA‐proved HGPS drug that can be used clinically (Dhillon, 2021). It is a farnesylation inhibitor that can disrupt the post‐translational modification of progerin. However, Lonafarnib inhibits the farnesylation of proteins other than progerin and triggers severe side effects in cells and patients (Arnold et al., 2021; Yang et al., 2010). We and others also showed that Lonafarnib decreases the proliferation of HGPS cells (Figure 1l,m and ref *31*). In addition, the effect of Lonafarnib on delaying physiological aging is questionable as the role of progerin in physiological aging has yet to be established (Primmer et al., 2022). On the contrary, mitophagy activator UMI‐77 exhibits no significant toxicity in HGPS cells or mice (Figure 1l,m and Figure S6G–K). Furthermore, UMI‐77 promotes the proliferation capacity of HGPS cells and presents antiaging effects toward both HGPS mice and middle‐aged wild‐type mice. These results indicate that mitophagy induction is a safer intervention for HGPS, corroborate previous findings that mitophagy plays a role in aging (Fang et al., 2019; Girotra et al., 2023; Ryu et al., 2016), and provide a novel mitophagy activator for antiaging intervention. Several questions remained to be elucidated on the antiaging interventions by UMI‐77. Firstly, whether UMI‐77 could directly ameliorate aging‐associated phenotypes in aging mice remained to be tested. In addition, thorough toxicity tests in mice are required to evaluate the safety of long‐term UMI‐77 treatment.

Mechanistically, our transcriptome results showed that mitophagy induction modulates aging hallmarks by significantly suppressing the STING‐NF‐κB pathway, which is the classical pathway regulating inflammation, and consequentially inhibiting the SASP expression in late‐passage HGPS‐MSCs. Previous studies reported that reducing inflammation and SASP secretion in the premature aging model can ameliorate symptoms of aging (Coll‐Bonfill et al., 2020; Liu et al., 2019; Squarzoni et al., 2021). Our study further supports the role of inflammation and SASP in driving premature aging and suggests that mitophagy induction is a promising strategy for alleviating detrimental inflammatory responses during physiological or pathological aging.

It is worth noting that although mitophagy induction has achieved satisfactory results in alleviating aging‐associated phenotypes in both cell and mouse models of HGPS, the level of progerin was not changed upon mitophagy induction (Figure S6L). It will be interesting to explore whether a combined treatment strategy of mitophagy induction with other treatments targeting progerin, such as ASO and CRISPR‐mediated base‐editing (Erdos et al., 2021; Koblan et al., 2021), can achieve synergetic effects on HGPS treatment. On the contrary, although we showed that HGPS cells exhibited defective mitophagy, it remains to be elucidated how progerin affects mitophagy in HGPS diseases. A recent study showed that progerin affected the nucleocytoplasmic shuttling of p300 in HGPS cells and consequentially inhibited autophagy through p300‐mediated acetylation on mTORC1 (Son et al., 2024). It is plausible that progerin impairs mitophagy through similar mechanisms.

## AUTHOR CONTRIBUTIONS

X.F., Z.L., and H.X. conceived the project; Y.S., L.X., and X.C. designed the experiments; Y.S., L.X., Y.L., Z.C., and Y.X. performed the experiments. S.J. performed bioinformatics analyses. Y.S. and X.F. wrote the manuscript. Y.L., G.W., J.W., N.S., L.H., J.Z., J.M., H.X., and Z.L. reviewed and revised the manuscript. All authors were involved in the interpretation of data.

## CONFLICT OF INTEREST STATEMENT

The authors declare no competing interests.

## Supporting information


Figures S1–S6.

## Data Availability

All data are available in the main text or the supplementary materials.
