# Supplementary material for: Mitophagy defect mediates the aging‐associated hallmarks in Hutchinson–Gilford progeria syndrome
Source: Aging Cell. 2024 Mar 14;23(6):e14143. doi: 10.1111/acel.14143 (PMC11296130; doi:10.1111/acel.14143)
Supplement: Supplementary file 1 — Figures S1–S6. [file ACEL-23-e14143-s001.docx]

**Supplementary Figures**

**Fig. S1 | (Related to Fig.1)
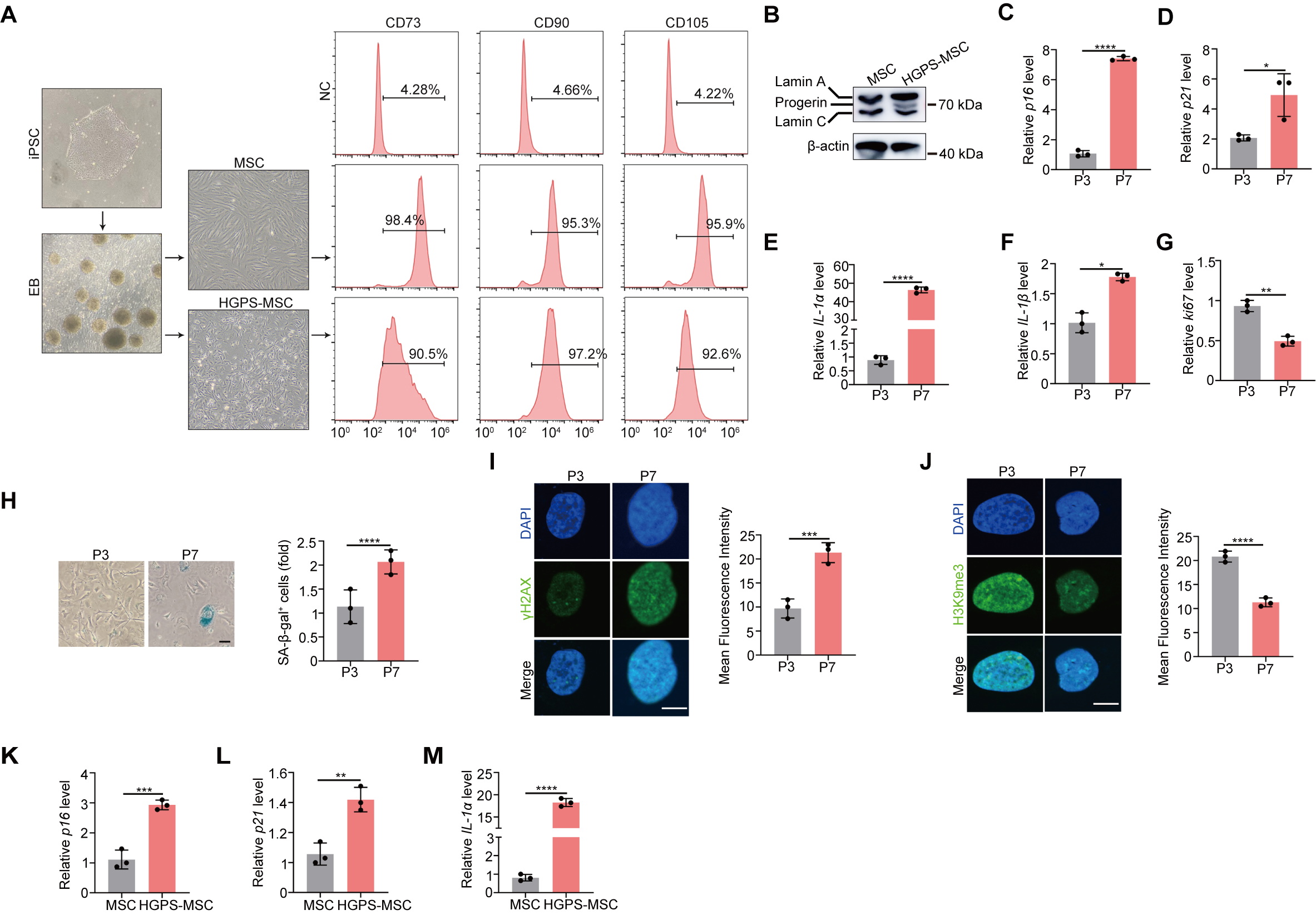
**

**Fig. S1. HGPS-MSCs exhibit augmented aging hallmarks compared to wild-type MSCs.**

(**A**) Flow cytometry analysis of CD73, CD90, and CD105 expression in iPSC-derived MSCs or HGPS-MSCs.

(**B**) Western blot analysis of lamin A/C and progerin in MSCs and HGPS-MSCs at passage 7.

(**C-G**) q-PCR analysis of *p16, p21, IL-1α, IL-1β*, and *KI67* mRNA expression in early-passage and late-passage HGPS-MSCs.

(**H**) SA-β-gal staining and quantification of early-passage and late-passage HGPS-MSCs. Scale bars, 20 μm.

(**I-J**) Immunofluorescence analysis and quantification of γH2AX and H3K9me3 in early-passage and late-passage HGPS-MSCs. Scale bars, 20 μm.

(**K-M**) q-PCR analysis of *p16, p21*, and *IL-1α* mRNA expression in MSCs and HGPS-MSCs at P7. Scale bars, 20 μm.

Data are presented as the mean ± s.d. Unpaired t-test was used for statistical analysis. n = 3 biological repeats. **P* < 0.05, ***P* < 0.01, ****P* < 0.001, *****P* < 0.0001.

**Fig. S2 | (Related to Fig. 1-2)**


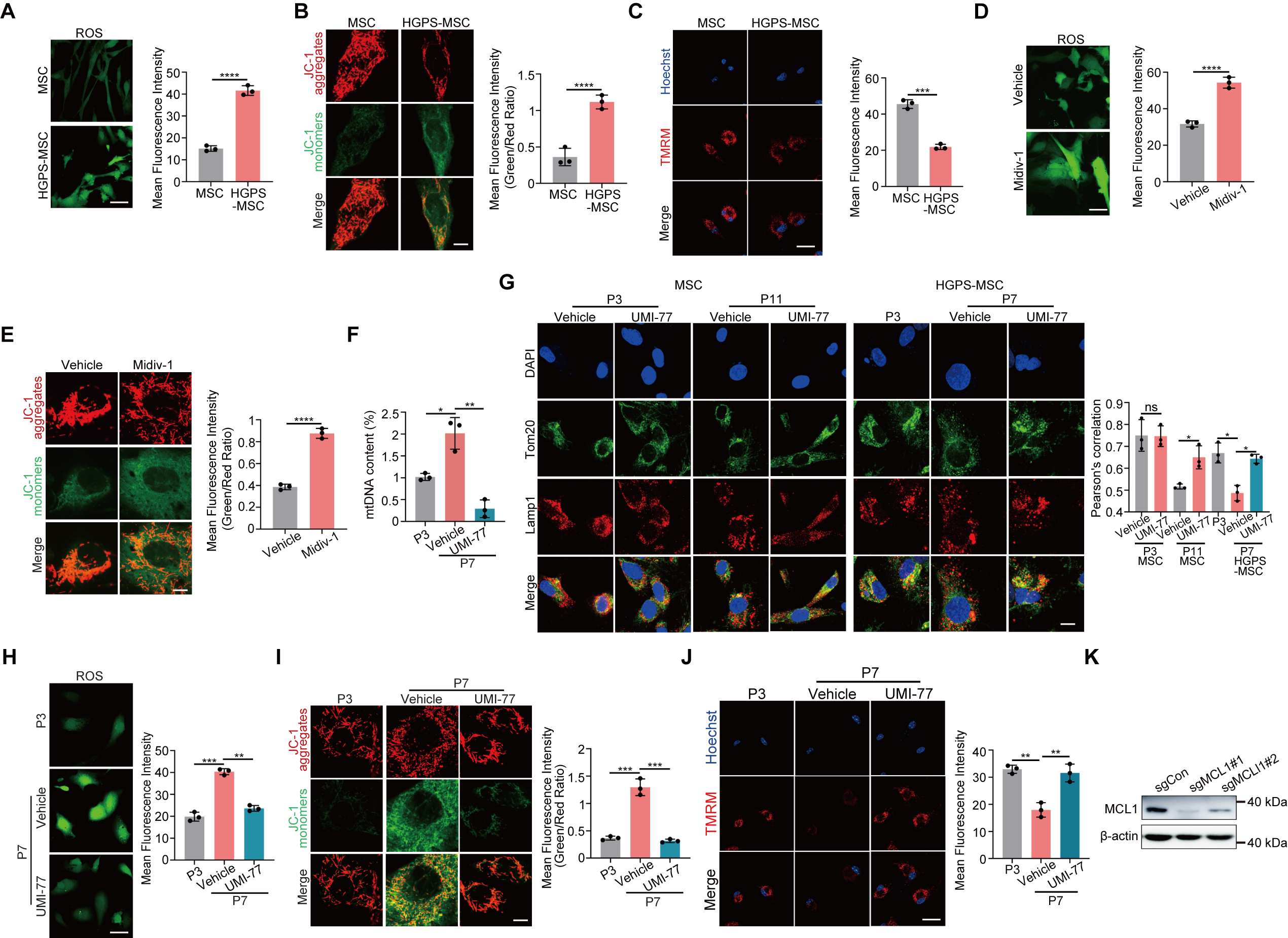


**Fig. S2. Mitophagy defects mediate mitochondria dysfunction in HGPS-MSCs.**

(**A**) Immunofluorescence analysis and quantification of DCFH-DA-based ROS in MSCs and HGPS-MSCs at P7. Scale bars, 20 μm.

(**B-C**) The mitochondrial membrane potential was detected by JC-1 **(B)** or TMRM **(C)** in MSCs and HGPS-MSCs at P7. Scale bars, 20 μm.

(**D**) Immunofluorescence analysis and quantification of DCFH-DA-based ROS in HGPS-MSCs treated with or without Midiv-1. Scale bars, 20 μm.

(**E**) The mitochondrial membrane potential was detected by JC-1 in HGPS-MSCs treated with or without Midiv-1. Scale bars, 20 μm.

(**F**) q-PCR analysis of mtDNA content in early passage HGPS-MSCs (P3), late passage HGPS-MSCs (P7), and UMI-77-treated late passage HGPS-MSCs.

(**G**) Co-staining Lamp1 and Tom20 in MSCs and HGPS-MSCs treated with or without UMI-77 and Pearson coefficient are quantified. Scale bars, 20 μm.

(**H**) Immunofluorescence analysis and quantification of DCFH-DA-based ROS in passage 7 (P7) HGPS-MSCs treated with or without UMI-77 (1 μM). Scale bars, 20 μm.

(**I-J**) The mitochondrial membrane potential was detected by JC-1 **(I)** or TMRM **(J)** in passage 7 (P7) HGPS-MSCs treated with or without UMI-77 (1 μM). Scale bars, 20 μm.

(**K**) Western blot analysis confirms the perturbation efficiency of MCL1.

Data are presented as the mean ± s.d. Unpaired t-test was used for statistical analysis. n = 3 biological repeats. **P* < 0.05, ***P* < 0.01, ****P* < 0.001, *****P* < 0.0001, ns, *P* > 0.05.

**Fig. S3 | (Related to Fig. 1-2)**

**
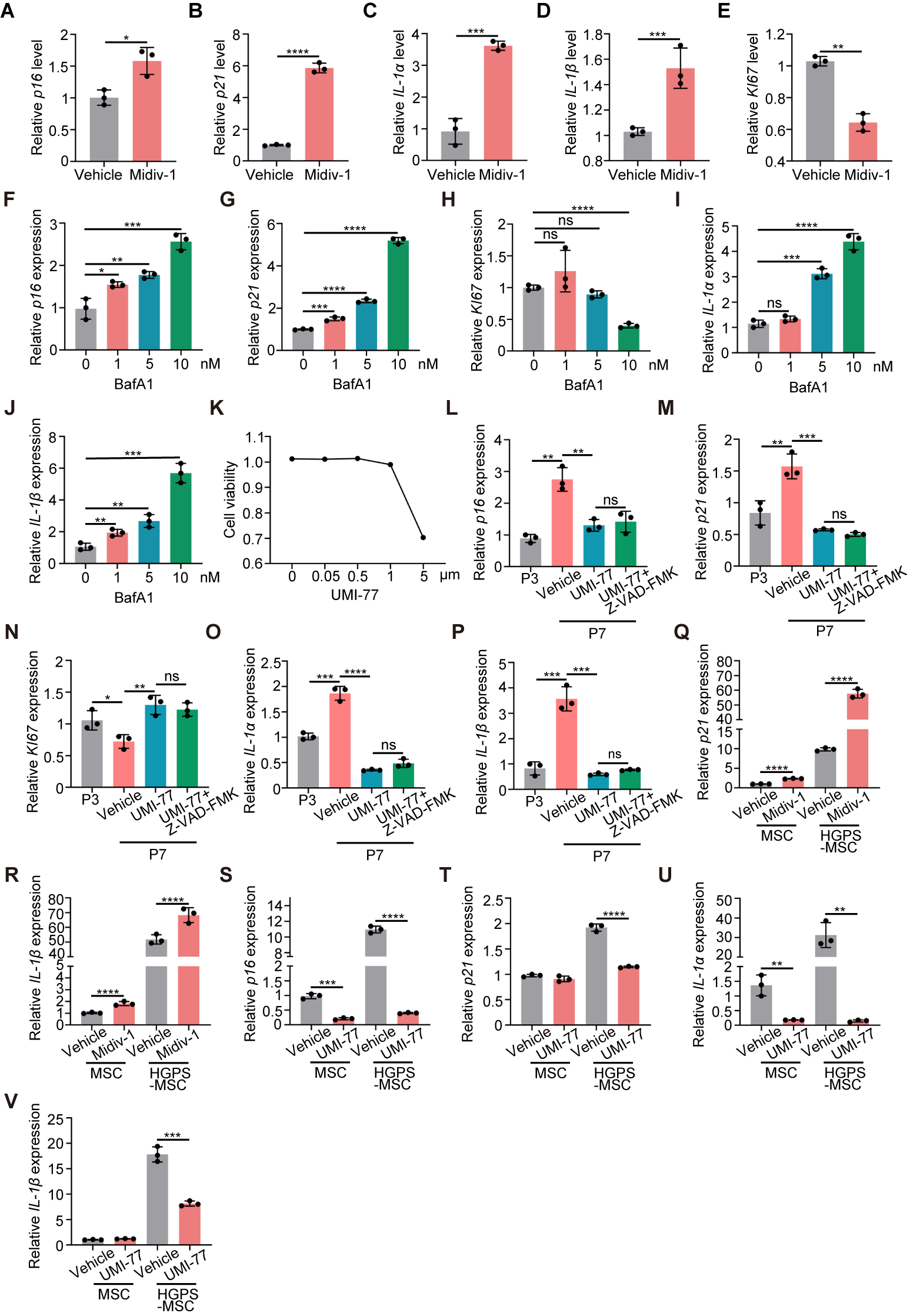
**

**Fig. S3. Mitophagy defects mediate cellular hallmarks associated with aging in HGPS-MSCs.**

(**A-E**) q-PCR analysis of *p16*, *p21*, *IL-1α*, *IL-1β*, and *KI67* mRNA expression in HGPS-MSCs treated with or without Midiv-1.

(**F-J**) q-PCR analysis of *p16*, *p21*, KI67, *IL-1α*, and *IL-1β* mRNA expression in HGPS-MSCs after being treated with different concentrations of bafilomycin A (BafA1).

(**K**) MTT assay to detect the effect of different concentrations of UMI-77 on HGPS-MSCS cell viability.

**(L-P)** q-PCR analysis of *p16*, *p21*, KI67, *IL-1α*, and *IL-1β* mRNA expression in HGPS-MSCs upon indicated treatment.

(**Q-R**) q-PCR analysis of *p21* and *IL-1β* mRNA expression in MSCs and HGPS-MSCs of the same passage (P3) upon indicated treatment. Midiv-1 (10 μM) was used.

**(S-V)** q-PCR analysis of *p16*, *p21*, *IL-1α*, and *IL-1β* mRNA expression in MSCs and HGPS-MSCs of the same passage (P7) upon indicated treatment. UMI-77 (1 μM) was used.

Data are presented as the mean ± s.d. Unpaired t-test was used for statistical analysis. n = 3 biological repeats. **P* < 0.05, ***P* < 0.01, ****P* < 0.001, *****P* < 0.0001, ns, *P* > 0.05.

**Fig. S4 | (Related to Fig. 2)**


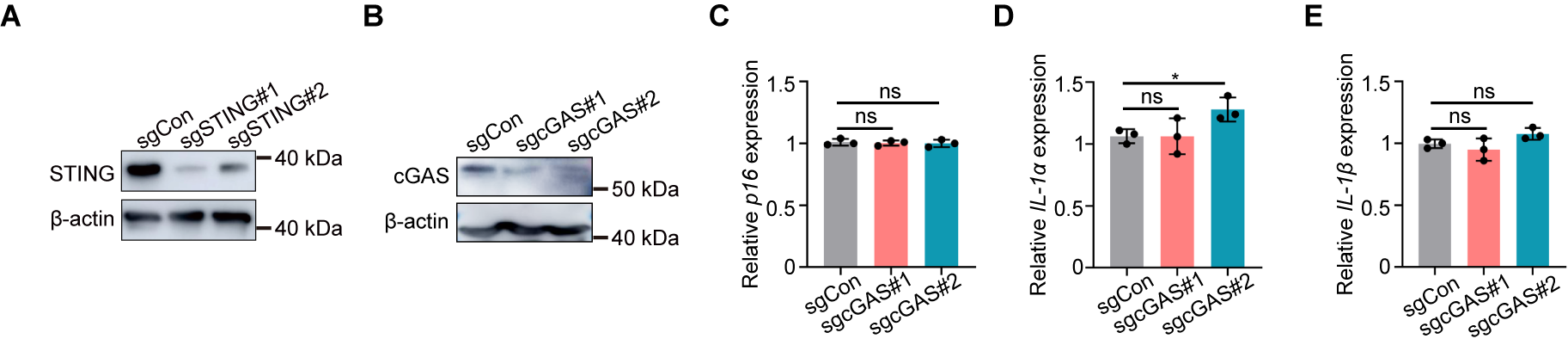


**Fig. S4. cGAS perturbation does not normalize the effect of UMI-77 on cellular aging-related phenotypes.**

(**A**) Western blot analysis confirms the perturbation efficiency of STING.

(**B**) Western blot analysis confirms the perturbation efficiency of cGAS.

(**C-E**) q-PCR analysis of *p16*, *IL-1α*, and *IL-1β* mRNA expression in UMI-77-treated HGPS-MSCs upon cGAS perturbation.

Data are presented as the mean ± s.d. Unpaired t-test was used for statistical analysis. n = 3 biological repeats. **P* < 0.05, ns, *P* > 0.05.

**Fig. S5 | (Related to Fig. 3)**


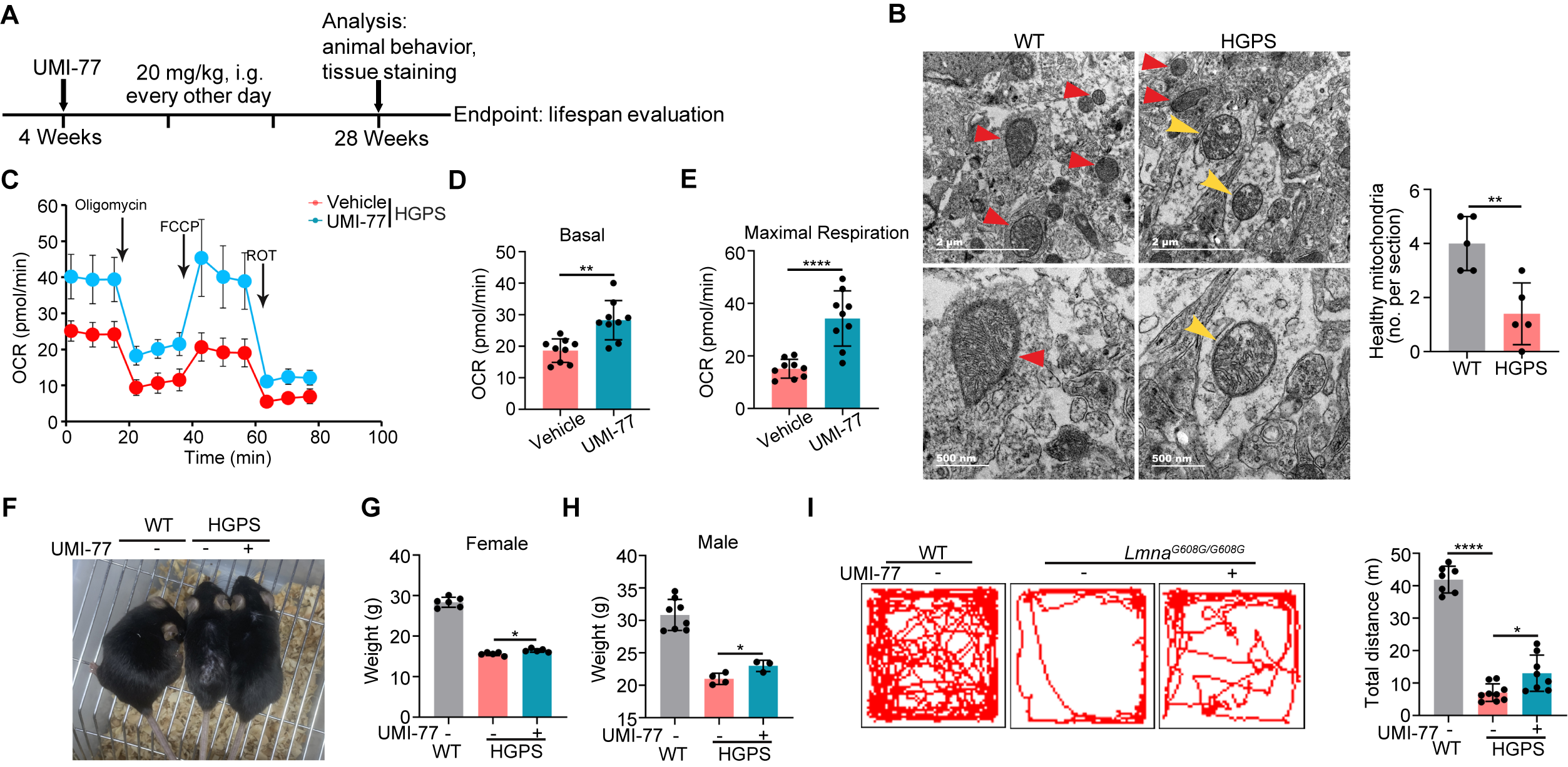


**Fig. S5. UMI-77 administration restores mitochondria function and improves the health of *Lmna^G608G/G608G^ mice.***

**(A)** Schematic depiction of the UMI-77 administration plan in HGPS mice.

**(B)** Representative TEM images (left) and quantification (right) of healthy mitochondria numbers in the brain of WT and HGPS mice. n = 3.

**(C-E)** Cellular oxygen consumption rates (OCR) in liver cells from 6-month HGPS mice treated with or without UMI-77. Basal respiration and maximal respiratory capacity are quantified in (D) and (E), respectively. n = 9 biological repeats.

(**F**) Images of wild-type male mice (WT), and *Lmna^G608G/G608G^* male mice treated with or without UMI-77.

(**G-H**) Bodyweight of WT, and *Lmna^G608G/G608G^* mice treated with or without UMI-77. For female mice, n = 6 (WT), 5 (HGPS), and 5 (HGPS with UMI-77). For male mice, n = 8 (WT), 4 (HGPS), and 3 (HGPS with UMI-77).

(**I**) The open field test showed the motion capabilities of WT (n = 7), and *Lmna^G608G/G608G^* male mice treated with (n = 8) or without UMI-77 (n = 9).

Data are presented as the mean ± s.d. Unpaired t-test was used for statistical analysis. **P* < 0.05, ***P* < 0.01, *****P* < 0.0001.

**Fig. S6 | (Related to Fig. 4)**


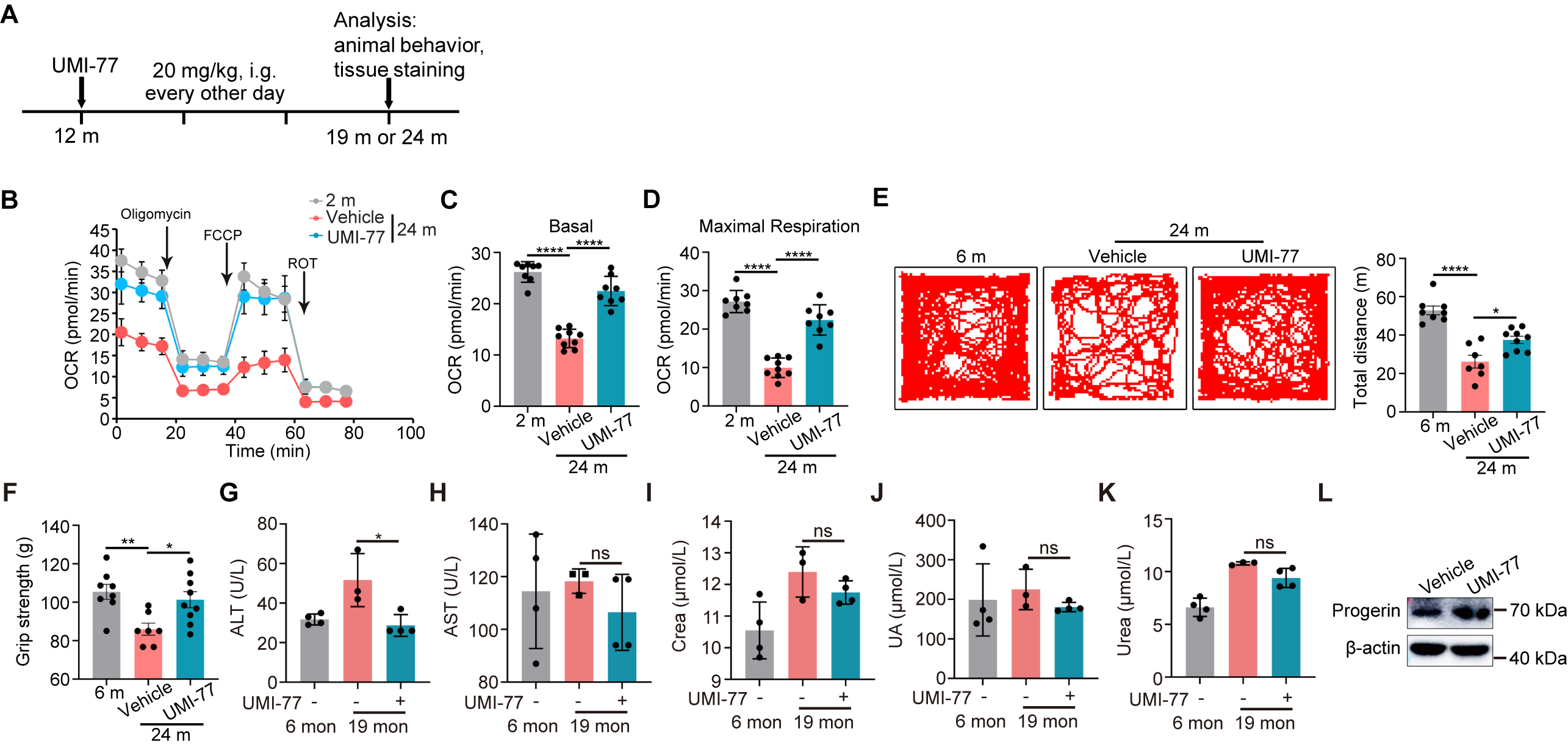


**Fig. S6. UMI-77 improves the health of wild-type aging mice.**

**(A)** Schematic depiction of the UMI-77 administration plan in wild-type mice.

**(B-D)** Cellular oxygen consumption rates (OCR) in 2-month (2 m) and 24-month (24 m) male mice treated with or without UMI-77. Basal respiration and maximal respiratory capacity are quantified in (B) and (C). n = 8 biological repeats.

**(E)** The open field test showed the motion capabilities of 6-month-old (6 m, n = 8) and 24-month-old male mice treated with (24 m, n = 9) or without UMI-77 (n = 7).

**(F)** Forelimb grip strength analysis of 6-month-old (6 m, n = 8) and 24-month-old male mice treated with (24 m, n = 9) or without UMI-77 (n = 7).

(**G-K**) The serum levels of alanine aminotransferase (ALT), aspartate aminotransferase (AST), creatinine (Crea), uric acid (UA), and urea in 6-month-old (6 mon, n = 4) and 19-month-old male mice treated with (19 mon, n = 4) or without UMI-77 (n = 3).

(**L**) Western blot analysis of progerin in HGPS-MSC treated with or without UMI-77. n = 3.

Data are presented as the mean ± s.d. Unpaired t-test was used for statistical analysis. *P* < 0.05, ***P* < 0.01, *****P* < 0.0001, ns, *P* > 0.05.
